# Do psychiatric decision units make a difference? An analysis from a liaison psychiatry service in Greater London

**DOI:** 10.1192/j.eurpsy.2023.236

**Published:** 2023-07-19

**Authors:** P. Walters, J. H. Tan, L. Premalatha, J. Adejumo

**Affiliations:** Croydon Liaison Psychiatry, South London and Maudsley NHS Foundation Trust, London, United Kingdom

## Abstract

**Introduction:**

CAU (Clinical assessment unit) was developed at Croydon University Hospital (CUH) in conjunction with the South London and Maudsley (SLaM) mental health trust in response to the Covid19 pandemic to relieve pressure on services in A&E (Accident and Emergency) and to support the already existing need to provide a more clinically appropriate space for patients presenting to A&E with acute mental health concerns. The clinical model for the unit was developed with input from service users, SLaM, CUH and CCG (Clinical Commissioning Group); and was fully established in October 2021 within a month of conception. CAU is located within close proximity to A&E which is convenient and incurs no transfer cost. Similar units have been developed internationally to address similar concerns *(Goldsmith et al 2021, Wiley online library 2021 12849)*

**Objectives:**

To evaluate the financial and clinical impact of the clinical assessment unit after one year in operation

**Methods:**

This is a cross sectional study, data was collected from EPJs Reports (SLaM’s patient data reporting system), excel spread sheet collecting data based on referrals to the service over the one-year period from 8/9/2021 – 5/10/2022. This included a trial period from September 2021 – October 2021 where the service was running at half capacity.

CAU is open to capacitous adults aged 18-65 presenting to A&E. Exclusion criteria: individuals conveyed by the police, those under MHA, on-going physical health concerns, diagnosis of learning disability with no primary mental health need, diagnosis of dementia and homelessness

**Results:**

3,322 patients were referred to the Liaison service and of those 402 or 12% of those patients to A&E were transferred to CAU

The 402 service users over the period of one year spent a total of 11, 351 hours in CAU

The main diagnosis of patient admitted to CAU fell into the diagnostic categories F30-39 43.5 %, and F60-69 27%.

The majority of patients were admitted awaiting informal admission and 1/3 of plans for discharge destination were made on CAU. 10 % of patient were discharged on a least restrictive outcome, which has cost benefits for acute mental health trust. This one-year period showed cost saving of £462,112 for 24 hours stay in ED with support staff.

**Image:**

**Figure:**
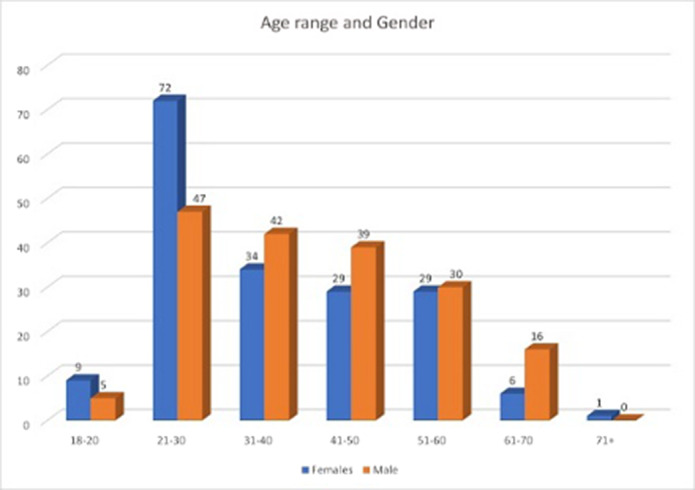

**Image 2:**

**Figure:**
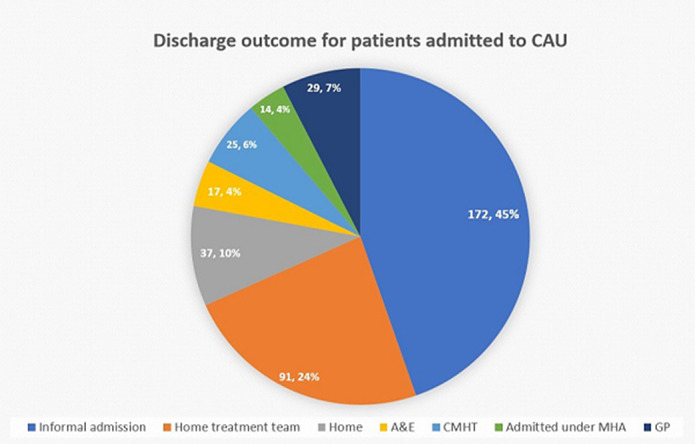

**Conclusions:**

CAU offers the opportunity to engage and re-assess service users to allow consideration of least restrictive options for on-going care. CAU has financial benefits in way of saving cost on time spent in ED awaiting review and cost for agency staff to provide 1:1 support.

The success of CAU has led to collaboration in the developpment of other acute services in London including that of the Recovery Space which offers community support for service users following discharge from hospital after an acute mental health crisis. Service user feedback has been positive and reflects the importance of the service and its suitability for its target group.

**Disclosure of Interest:**

None Declared

